# Choice of selectable marker affects recombinant protein expression in cells and exosomes

**DOI:** 10.1016/j.jbc.2021.100838

**Published:** 2021-05-27

**Authors:** Chenxu Guo, Francis K. Fordjour, Shang Jui Tsai, James C. Morrell, Stephen J. Gould

**Affiliations:** Department of Biological Chemistry, School of Medicine, Johns Hopkins University, Baltimore Maryland, USA

**Keywords:** antibiotics, cell culture, exosomes (vesicles), extracellular vesicle, tetraspanin, recombinant protein expression, selectable markers, transgenic, CTCS, conditioned tissue culture supernatant, DMEM, Dulbecco’s modified Eagle’s medium, DPBS, Dulbecco’s modified phosphate buffered saline, EBNA1, EBV nuclear antigen 1, EBV, Epstein–Barr virus, NLS, nuclear localization signal, NTA, nanoparticle tracking analysis, WHP, Woodchuck hepatitis (WHP), WPRE, WH virus posttranscriptional regulatory element

## Abstract

Transgenic mammalian cells are used for numerous research, pharmaceutical, industrial, and clinical purposes, and dominant selectable markers are often used to enable the selection of transgenic cell lines. Using HEK293 cells, we show here that the choice of selectable marker gene has a significant impact on both the level of recombinant protein expression and the cell-to-cell variability in recombinant protein expression. Specifically, we observed that cell lines generated with the *NeoR* or *BsdR* selectable markers and selected in the antibiotics G418 or blasticidin, respectively, displayed the lowest level of recombinant protein expression as well as the greatest cell-to-cell variability in transgene expression. In contrast, cell lines generated with the *BleoR* marker and selected in zeocin yielded cell lines that expressed the highest levels of linked recombinant protein, approximately 10-fold higher than those selected using the *NeoR* or *BsdR* markers, as well as the lowest cell-to-cell variability in recombinant protein expression. Intermediate yet still-high levels of expression were observed in cells generated with the *PuroR*- or *HygR*-based vectors and that were selected in puromycin or hygromycin, respectively. Similar results were observed in the African green monkey cell line COS7. These data indicate that each combination of selectable marker and antibiotic establishes a threshold below which no cell can survive and that these thresholds vary significantly between different selectable markers. Moreover, we show that choice of selectable marker also affects recombinant protein expression in cell-derived exosomes, consistent with the hypothesis that exosome protein budding is a stochastic rather than determinative process.

The creation of transgenic mammalian cell lines was pioneered in the 1980s by Berg and colleagues (1, 2, 3, 4). In general, this process involves transfecting or transducing cells with a recombinant DNA vector that carries the gene of interest and a selectable marker gene and then selecting for transgene-expressing cells using an appropriate antibiotic (3, 4). This approach has been widely employed, has led to the creation of many useful transgenic cell lines, and is still in use today. However, many of the antibiotic-resistant cell clones generated by this approach express low or undetectable levels of the linked transgene (5). As a result, experiments that require high-level expression of the transgene of interest often require the isolation, expansion, and screening of dozens (or more) single-cell clones (SSCs) before one can obtain a cell line that displays the desired level of transgene expression.

Many researchers have devoted significant time, effort, and resources to improving the outcomes of mammalian cell transgenesis experiments. Much of this effort has been directed at improved vector design, resulting in the identification of many *cis*-acting features that affect transgene expression, including transcriptional regulatory regions (6, 7, 8, 9, 10), mRNA polyadenylation (pAn) sites (11), introns (12), mRNA export and/or translation signals (*e.g.*, the Woodchuck hepatitis virus [WHP] posttranscriptional regulatory element [WPRE] (13)), and *cis*-acting inhibitors of gene silencing (10, 14). Other studies have revealed that transgene expression can be improved by eliminating or reducing bacterial or viral sequences that may induce transgene silencing (15, 16), such as by use of DNA minicircles (17) or DNA transposons (*e.g.*, Sleeping Beauty (18, 19) or PiggyBac (20, 21)). Transgene delivery can also be achieved *via* replicating episomes, as plasmids carrying Epstein–Barr virus (EBV) origin of replication (OriP) and the EBV nuclear antigen 1 gene (EBNA1) may boost transgene expression *via* elevated gene dosage effects (22). Researchers have also invented technologies that direct transgene integration into sites of the host cell chromosome that are compatible with high-level, stable transgene expression, such as recombinase-mediated cassette exchange (23), phage ΦC31-mediated DNA integration (24), and CRISPR/Cas9-mediated genome editing (25). Although these site-directed insertional strategies represent a significant technological advance, several are limited to specific, previously engineered recipient cell lines, all require the isolation, expansion, and characterization of numerous SCCs, and none are designed specifically for eliciting the very highest levels of transgene expression.

While these and other studies have led to significant improvements and advanced the field of mammalian cell transgenesis, none have systematically interrogated the relative effectiveness of the available dominant selectable markers. The five dominant selectable markers in widespread are the *NeoR*, *BsdR*, *HygR*, *PuroR*, and *BleoR* genes, which confer resistance to the selective antibiotics G418/geneticin, blasticidin, hygromycin B, puromycin, and zeocin, respectively (3, 26, 27, 28, 29). It is therefore unclear whether the choice of selectable marker has any predictable effects on the outcome of mammalian cell transgenesis experiments. This report shows that the choice of selectable marker and the process of antibiotic selection has a significant effect on the expression of linked recombinant proteins. Furthermore, many of the experiments in this paper addressed the effects of selectable marker on expression of a recombinant exosomal cargo protein, CD81, so that our results might have relevance to the engineering of exosomes. Exosomes are small, extracellular vesicles (sEVs) of ~30–150 nm in diameter that are released by all human cell types, can transmit signals and molecules to other cells in a pathway of intercellular vesicle traffic, and are of increasing use as delivery vehicles for vaccines and therapeutics (30).

## Results

### Heterogeneous expression from NeoR and BsdR-linked transgenes

**Figure 1:**
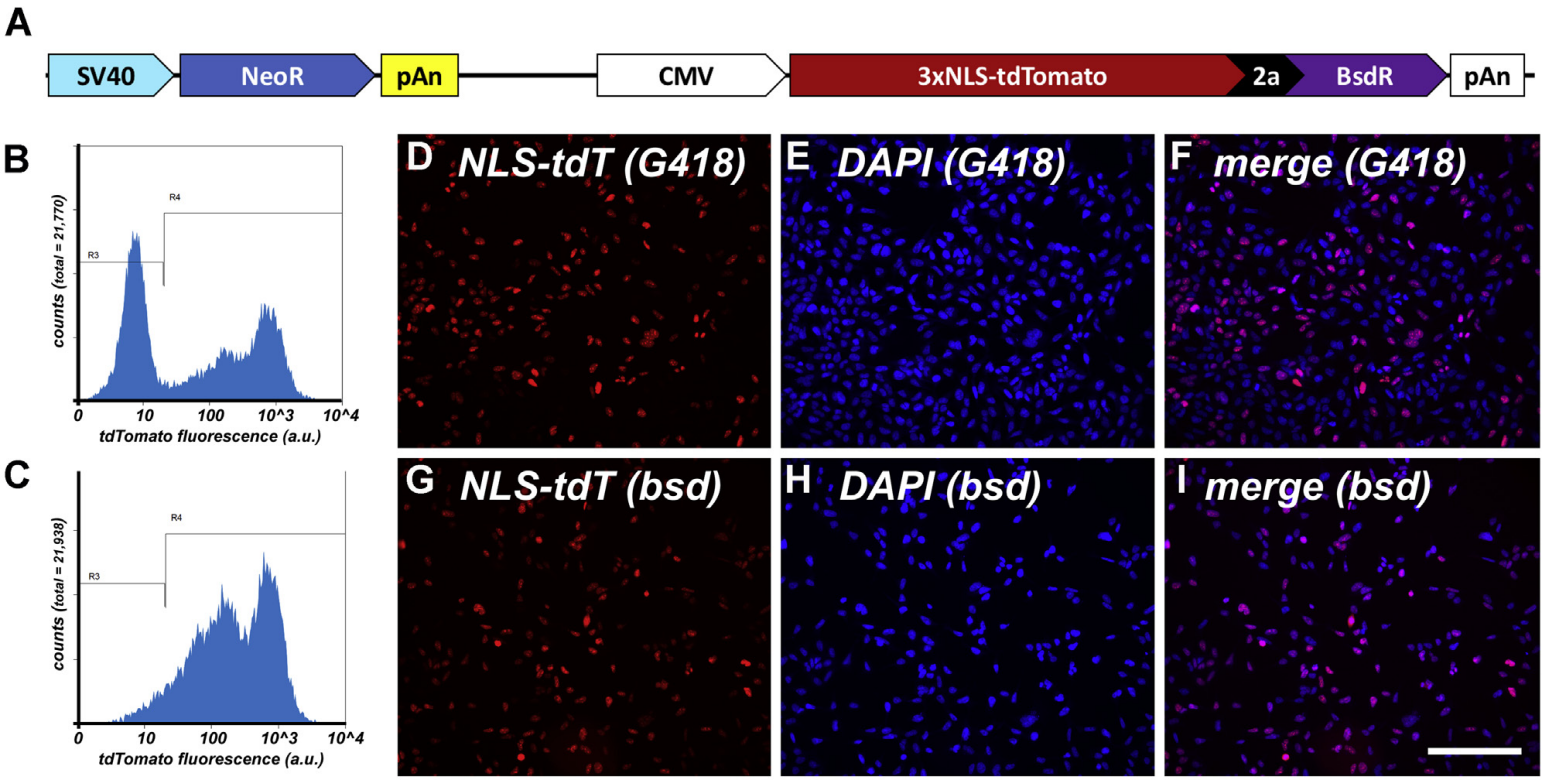
**Transgene expression profiles of HEK293 cell lines arising from two-gene and bicistronic selections.***A*, line diagram showing the NeoR and 3xNLS-tdTomato-2a-BsdR transgenes of the plasmid pJM825. *B* and *C*, flow cytometry scatter plots of HEK293 cells that had been transfected with pJM825 and then selected in (*B*) G418 or (*C*) blasticidin for 4 weeks. Numbers of cells are shown on the *y*-axis while relative fluorescent brightness (arbitrary units (a.u.)) is shown on the *x*-axis (log scale). R3 shows the experimentally determined background fluorescence of HEK293 control cells, whereas R4 denotes red fluorescence above background. *D–I*, fluorescence micrographs of DAPI-stained (D–F) HEK293/pJM825/G418-resistant cells and (*G–I*) HEK293/pJM825/blasticidin-resistant cells, showing (*D*, *G*) 3xNLS-tdTomato, (*E*, *H*) DAPI, and (*F*, *I*) merged images. Bar: 100 μm. These experiments were performed in triplicate.

HEK293 cell lines are commonly used for biochemical and cell biological studies and are an approved cell factory for producing biological materials and drugs (5, 31). To document the results of a classic plasmid-based mammalian cell transgenesis experiment (4, 5), we transfected HEK293 cells with pcDNA3-3xNLS-tdTomato-2a-BsdR, which carries two independent functional genes. One is designed to express the *NeoR* gene from the SV40 early promoter, and the other is designed to express a bicistronic ORF encoding (i) 3xNLS-tdTomato, a form of the red fluorescent protein tdTomato (32) that carries three copies of a nuclear localization signal (NLS) (33) at its N-terminus, (ii) the porcine *Teschovirus* 2a peptide (34), and (iii) the selectable marker blasticidin deaminase (29). (Fig. 1*A*). Two days after transfection, the cells were placed into complete media containing either 400 ug/ml G418 to select for G418-resistant cell lines or 20 ug/ml blasticidin to select for blasticidin-resistant cell lines. Two weeks later, the cells were pooled and examined by flow cytometry to determine the range of 3xNLS-tdTomato expression in each cell population (Fig. 1, *B* and *C*). Assaying thousands of cells from these polyclonal cell lines revealed that ~50% of cells in the G418-resistant line cells lacked detectable levels of 3xNLS-tdTomato expression. This is not surprising, given that the 3xNLS-tdTomato gene represents a sizeable proportion of the transfected plasmid and will therefore be disrupted in a significant proportion of G418-resistant cell lines due to the random nature of plasmid linearization that occurs during transgene insertion into host chromosomes (4, 5). In contrast, the blasticidin-resistant cell line contained a far lower percentage of nonexpressing cells, consistent with the fact that this transgene enforces a 1:1 stoichiometry between 3xnNLS-tdTomato expression and blasticidin deaminase enzymes expression. However, this cell line also displayed a pronounced cell-to-cell variability in 3xNLS-tdTomato expression, demonstrating that resistance to BsdR showed no correlation to any particular level of transgene expression. These conclusions were also apparent from fluorescence microscopy experiments (Fig. 1, *D*–*I*).

### Choice of selectable marker affects recombinant protein expression

**Figure 2:**
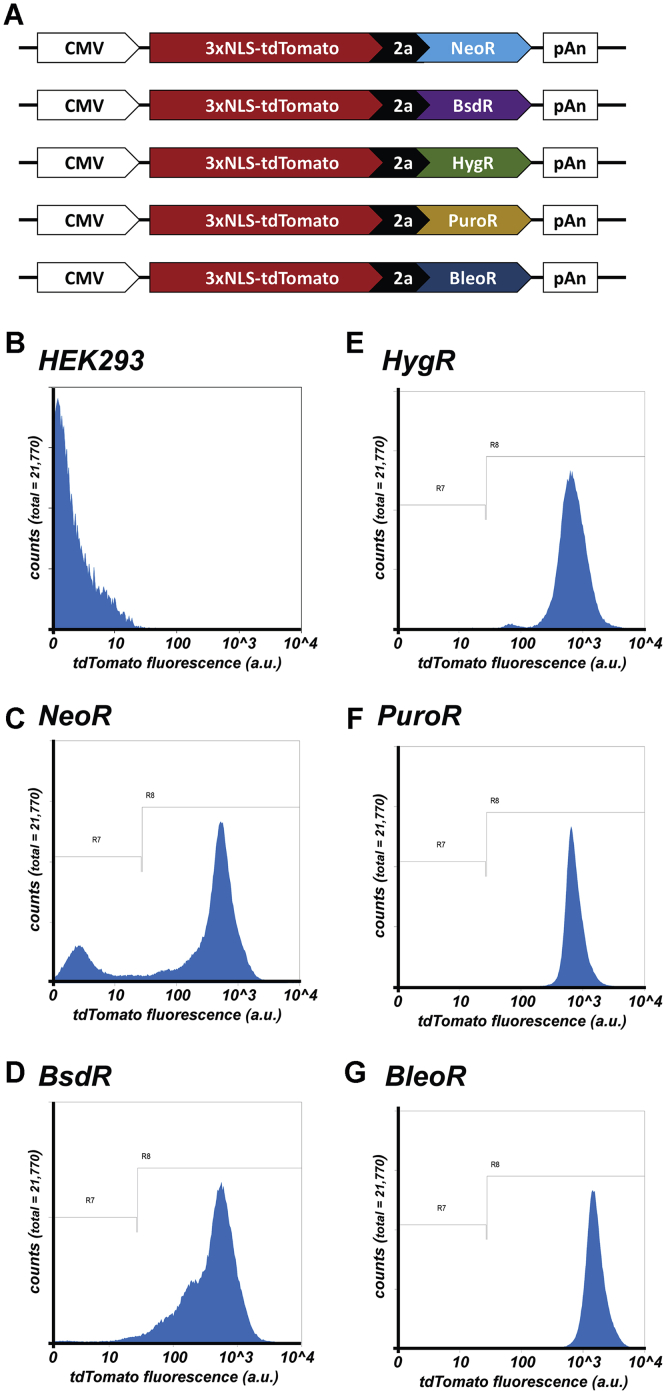
**Effect of selectable marker on linked expression of 3xNLS-tdTomato**. *A*, line diagram of transgenes encoding 3xNLS-tdTomato, the viral p2a peptide, and the *NeoR*, *BsdR*, *HygR*, *PuroR*, and *BleoR* selectable markers (not to scale). Scatter plots of flow cytometric analyses of (*B*) HEK293 cells or (*C–G*) HEK293 cells transfected with plasmids encoding the above transgenes and selected for 4 weeks in media containing (*C*) G418, (*D*) blasticidin, (*E*) hygromycin, (*F*) puromycin, or (*G*) zeocin. Numbers of cells are shown on the *y*-axis while relative fluorescent brightness (arbitrary units (a.u.)) is shown on the *x*-axis (log scale). R7 shows the experimentally determined background fluorescence of HEK293 cells, whereas R8 denotes red fluorescence due to 3xNLS-tdTomato expression. These experiments were performed twice.

These results raised the question of whether all dominant selectable markers yield cell lines with similarly low and heterogeneous levels of recombinant protein expression. To explore this possibility, we created a new set of expression vectors designed to drive expression of a single gene comprised of a CMV promoter upstream of a bicistronic ORF that encoded 3xNLS-tdTomato separated by the p2a peptide from downstream NeoR (3) BsdR (29), HygR (27), PuroR (26), or BleoR (28) markers (Fig. 2*A*). HEK293 cells were transfected with each of these plasmids, grown for a day in normal media, and then incubated for 2 weeks in media containing G418, blasticidin, hygromycin, puromycin, or zeocin, respectively. Clones from each transfection were then pooled to generate five distinct cell lines, which were then examined for 3xNLS-tdTomato expression by flow cytometry (Fig. 2, *B*–*G*; Table 1). The NeoR- and BsdR-resistant cell lines displayed the lowest average relative brightness and high degrees of cell-to-cell variation in 3xNLS-tdTomato fluorescence (458, with a coefficient of variance (c.v.) = 103; and 522, with a c.v. = 82, respectively). In contrast, the HygR- and PuroR-based cell lines displayed higher and more homogeneous levels of 3xNLS-tdTomato expression (794, c.v. = 62; and 803, c.v. = 44, respectively), while the BleoR-based cell line displayed the highest and most homogeneous expression of all (1754, c.v. = 46).

**Table 1:** Flow cytometry data for cell lines expressing 3xNLS-tdTomato from nonreplicating plasmids

Cell line	Average relative brightness; c.v.	% nonexpressing cells
HEK293/pCJM-3xNLS-tdTomato^a^-2a-NeoR^a^	458; 103	22%
HEK293/pCJM-3xNLS-tdTomato^a^-2a-BsdR^a^	522; 82	3%
HEK293/pCJM-3xNLS-tdTomato^a^-2a-HygR^a^	794; 62	0.40%
HEK293/pCJM-3xNLS-tdTomato^a^-2a-PuroR^a^	803; 44	0.30%
HEK293/pCJM-3xNLS-tdTomato^a^-2a-BleoR^a^	1754; 46	0.20%
HEK293	3; 153	100%

a Codon optimized.

### Choice of selectable marker affects exosomal protein expression

**Figure 3:**
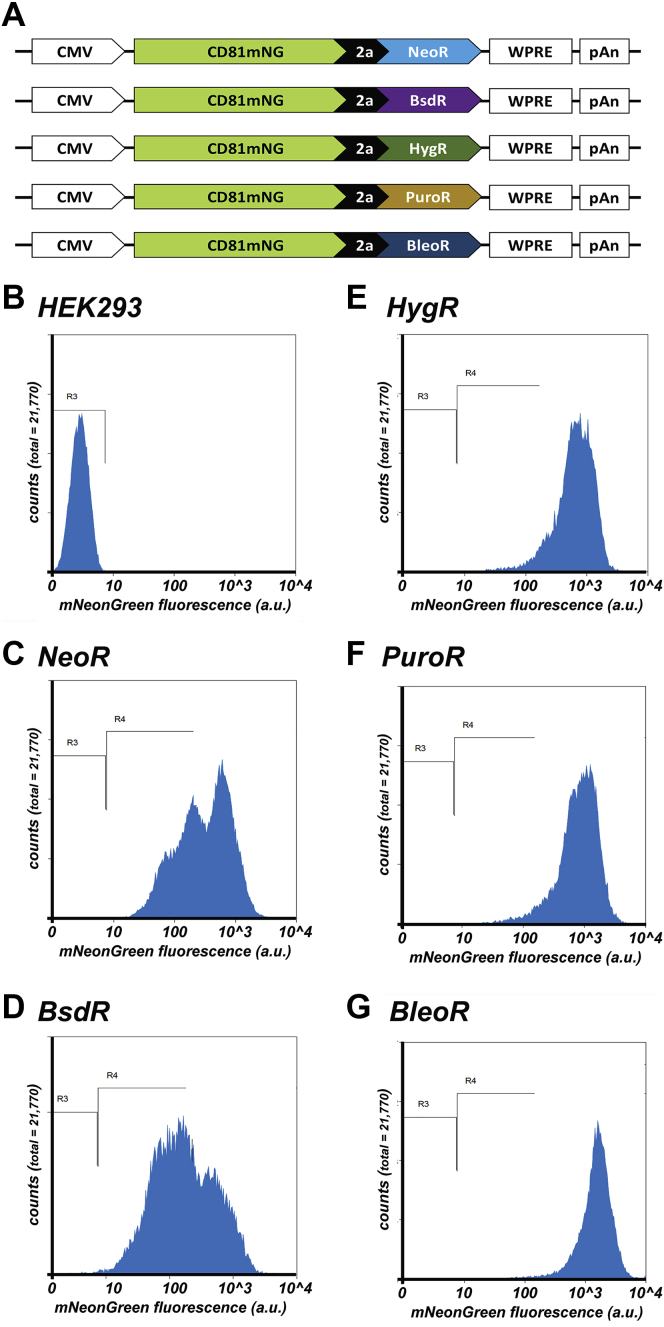
**Effect of selectable marker on linked expression of CD81-mNG.***A*, line diagram of transgenes encoding CD81mNG, the viral p2a peptide, and the *NeoR*, *BsdR*, *HygR*, *PuroR*, and *BleoR* selectable markers (not to scale). Scatter plots of flow cytometric analyses of (*B*) HEK293 cells or (*C–G*) HEK293 cells transfected with plasmids encoding the above transgenes and selected for 4 weeks in media containing (*C*) G418, (*D*) blasticidin, (*E*) hygromycin, (*F*) puromycin, or (*G*) zeocin. Numbers of cells are shown on the *y*-axis while relative fluorescent brightness (arbitrary units (a.u.)) is shown on the *x*-axis (log scale). R3 shows the experimentally determined background fluorescence of HEK293 cells, whereas R4 denotes green fluorescence due to CD81mNG expression. These experiments were performed twice.

**Figure 4:**
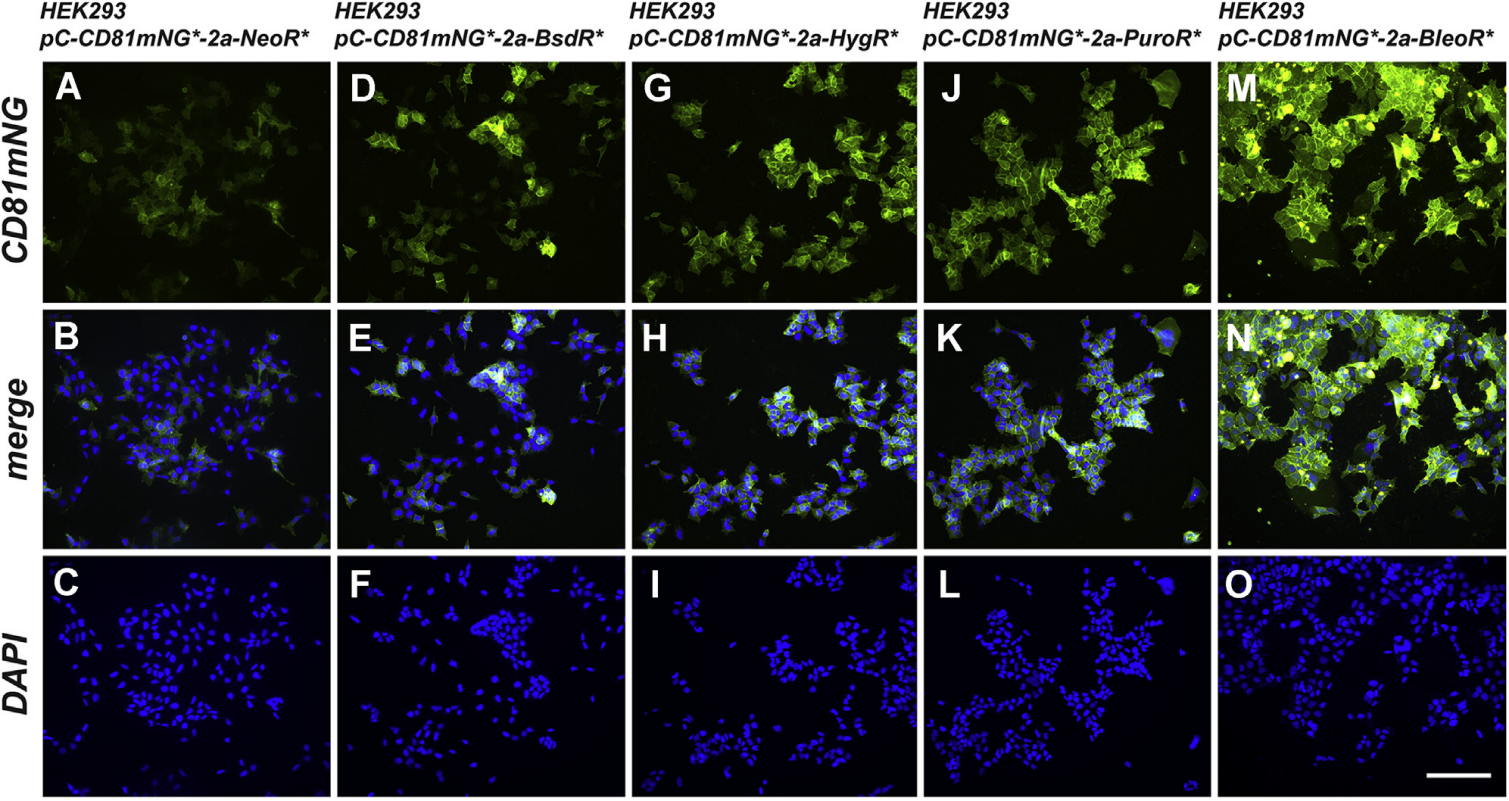
**Fluorescence micrographs of HEK293 cells transfected with CD81mNG-expressing transgenes**. HEK293 cells transfected with the five transgenes described in Figure 3*A* were selected for 4 weeks in (*A–C*) G418, (*D–F*) blasticidin, (*G–I*) hygromycin, (*J–L*) puromycin, or (*M–O*) zeocin, respectfully. Each of these five cell lines were then grown overnight on sterile cover glasses, fixed, stained with DAPI. Images show (*A*, *D*, *G*, *J*, *M*) mNeonGreen fluorescence, (*B*, *E*, *H*, *K*, *N*) DAPI fluorescence, and (*C*, *F*, *I*, *L*, *O*) the merge of the two. Bar: 100 μm. These experiments were performed in triplicate.

**Figure 5:**
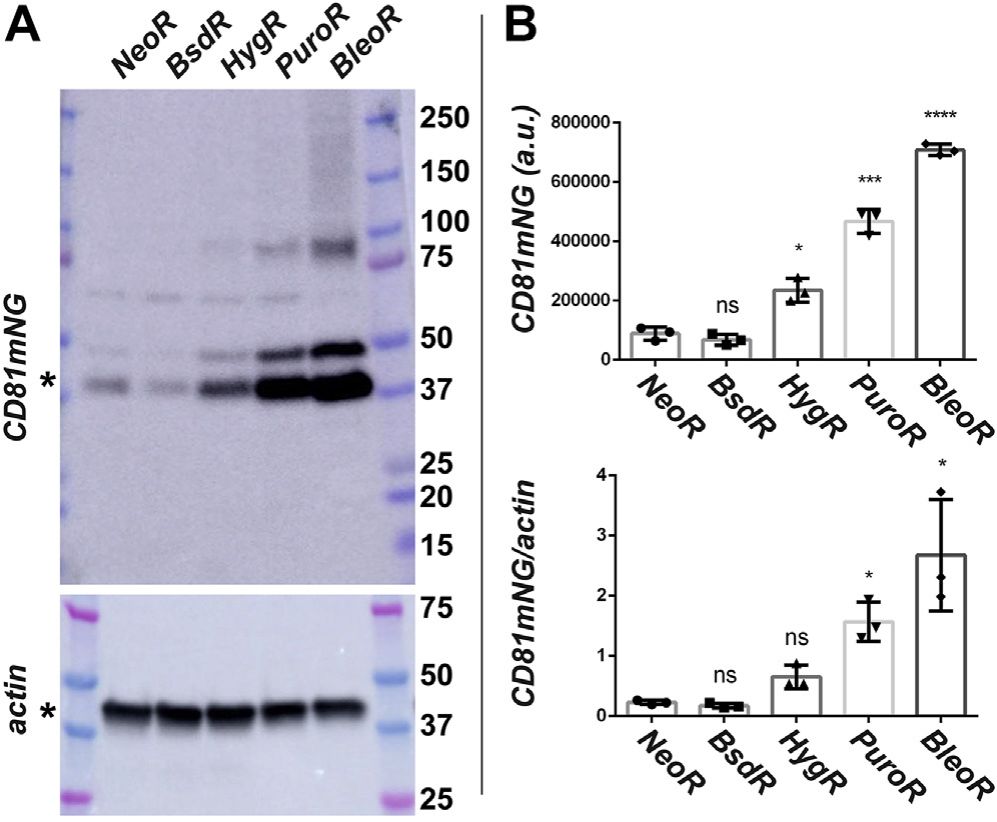
**Immunoblot analysis of HEK293 cells expressing CD81mNG**. HEK293 cells transfected with the five transgenes described in Figure 3*A* were selected for 4 weeks in G418, blasticidin, hygromycin, puromycin, or zeocin, respectfully. *A*, immunoblot analysis of cell lysates was probed using antibodies specific for (*upper panel*) the p2a tag (which is fused to the C-terminus of CD81mNG) and (*lower panel*) actin. MW markers are, from top, 250 kDa, 150 kDa, 100 kDa, 75 kDa (*pink*), 50 kDa, 37 kDa, 25 kDa (*pink*), 20 kDa, and 15 kDa. *B*, bar graphs show (*upper graph*) anti-2a signal intensity and (*lower graph*) the ratio of anti-2a immunoblot signals/actin immunoblot signals from three independent trials. Averages (bar height), standard error of the mean (error bars), and statistical significance (∗*p* ≤ 0.05; ∗∗∗*p* ≤ 0.0005; ∗∗∗∗*p* ≤ 0.00005) were calculated using Prism software and Welch's unequal variances *t* test. The raw data used in these analyses is also accessible (supporting information).

To determine whether these effects extended to other recombinant proteins, we created another set of vectors in which the 3xNLS-tdTomato coding region was replaced with that of CD81 (35), an integral plasma membrane protein that is highly enriched in exosomes (36), fused to green fluorescent protein mNeonGreen (37), allowing the detection of this protein (CD81mNG) by fluorescence-based techniques (Fig. 3*A*). HEK293 cells were transfected with these five plasmids, and the antibiotic-resistant clones were pooled to create five polyclonal cell lines. These lines were then examined by flow cytometry to measure the relative levels of CD81mNG fluorescence in thousands of cells within each population (Fig. 3, *B–G*; Table 2). The NeoR- and BsdR-derived cell lines once again displayed the lowest and most heterogeneous expression of their linked recombinant protein, with relative CD81mNG brightness levels of 465 (c.v. = 93) and 316 (c.v. = 126), respectively. In contrast, the cell lines derived by transfection with the *HygR*- and *PuroR*-based plasmids displayed higher and more homogeneous levels of expression <aid>(average relative CD81mNG brightness of 790 (c.v. = 63) and 1000 (c.v. = 63), respectively. Once again, the cells derived by transfection with the *BleoR*-based plasmid displayed the highest and most homogeneous levels of transgene expression (1749; c.v. = 55). Similar results were observed when these cell lines were interrogated by fluorescence microscopy (Fig. 4) or by immunoblot analysis (Fig. 5), the latter of which showed an approximately 10-fold increase in the expression of CD81mNG in the BleoR cell line relative to the *BsdR*-derived or *NeoR*-derived cell lines, with intermediate levels of expression in the *HygR* and *PuroR*-derived cell lines.

**Table 2:** Flow cytometry data for cell lines expressing CD81mNeonGreen from nonreplicating plasmids

Cell line	Average relative brightness; c.v.	% nonexpressing cells
HEK293/pC-CD81mNG^a^-2a-NeoR^a^	465; 93	0%
HEK293/pC-CD81mNG^a^-2a-BsdR^a^	316; 126	0%
HEK293/pC-CD81mNG^a^-2a-HygR^a^	790; 63	0.01%
HEK293/pC-CD81mNG^a^-2a-PuroR^a^	1000; 63	0.01%
HEK293/pC-CD81mNG^a^-2a-BleoR^a^	1749; 55	0.01%
HEK293	3; 37	100%

a Codon optimized.

### Genetic engineering of exosomes

**Figure 6:**
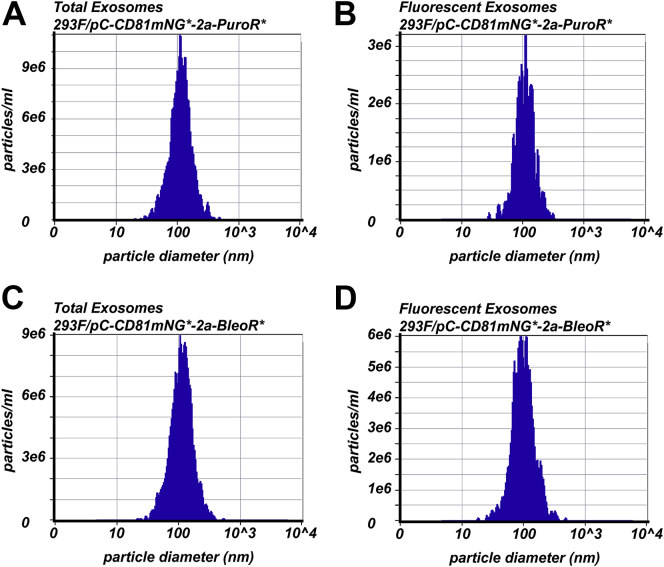
**Size distribution profiles of exosomes released by transgenic 293F cells.** Exosomes were collected from the tissue culture supernatants of (*A*, *B*) 293F/pC-CD81mNG-2a-PuroR and (*C*, *D*) 293F/pC-CD81mNG-2a-BleoR cell lines and assayed by nanoparticle tracking analysis. *A* and *B*, scatter plots of exosome concentration and size for (*A*) all 293F/pC-CD81mNG-2a-PuroR-derived exosomes and (*B*) green fluorescent 293F/pC-CD81mNG-2a-PuroR-derived exosomes. *C* and *D*, scatter plots of exosome concentration and size for (*C*) all 293F/pC-CD81mNG-2a-BleoR-derived exosomes and (*D*) green fluorescent 293F/pC-CD81mNG-2a-BleoR-derived exosomes. These experiments were performed once.

CD81 is among the most highly enriched exosomal proteins known (36) and has high potential as a carrier molecule for modifying exosome content. To determine whether its incorporation into exosomes is impacted by its level of expression in exosome-producing cells, we transfected 293F cells (a derivative of HEK293 cells) with the *PuroR*- and *BleoR*-linked CD81mNG expression vectors described above (pC-CD81mNG∗-2a-PuroR∗ and pC-CD81mNG∗-2a-BleoR∗). A day later we selected for antibiotic-resistant clones, which were expanded as pools of puromycin-resistant and zeocin-resistant cells. These two cell lines were inoculated into chemically defined media in shaker flasks and grown for 5 days. The cells were then removed from the conditioned and exosomes were purified by a combination of low speed centrifugation, size-exclusion filtration, filtration-based concentration, and size-exclusion chromatography. The two exosome preparations were then interrogated by nanoparticle tracking analysis (NTA) using a Particle Metrix Zetaview PMX220 (38) to measure the concentrations, sizes, and CD81mNG fluorescence of exosomes in each sample (Fig. 6; Table 3). Both preparations contained large numbers of extracellular vesicles, the vast majority of which had the size expected of exosomes (mean diameters of 112 nm and 114 nm, respectively), confirming that we had purified exosomes and not the larger, microvesicle class of extracellular vesicles. The puromycin-resistant cell line, which expresses high levels of CD81mNG, released a population of exosomes in which ~25% displayed detectable levels of CD81mNG fluorescence. In contrast, the zeocin-resistant cell line, which expresses higher levels of CD81mNG, released a population of exosomes in which 70% carried detectable levels of CD81mNG, a more than 2-fold increase in exosome occupancy, which is consistent with the stochastic model of exosome biogenesis (30).

**Table 3:** Exosome size, concentration, and fluorescence data as determined by NTA

Exosome-producing cell line	Number of exosomes	Average size
293F/pC-CD81mNG^a^-2a-PuroR^a^ (total)	3.8 × 10^11^	112 nm
293F/pC-CD81mNG^a^-2a-PuroR^a^ (fluorescent)	9.9 × 10^10^ (26%)	109 nm
293F/pC-CD81mNG^a^-2a-BleoR^a^ (total)	1.4 × 10^12^	114 nm
293F/pC-CD81mNG^a^-2a-BleoR^a^ (fluorescent)	9.8 × 10^11^ (70%)	97 nm

a Codon optimized.

### Consistent transgene expression across different platforms

**Figure 7:**
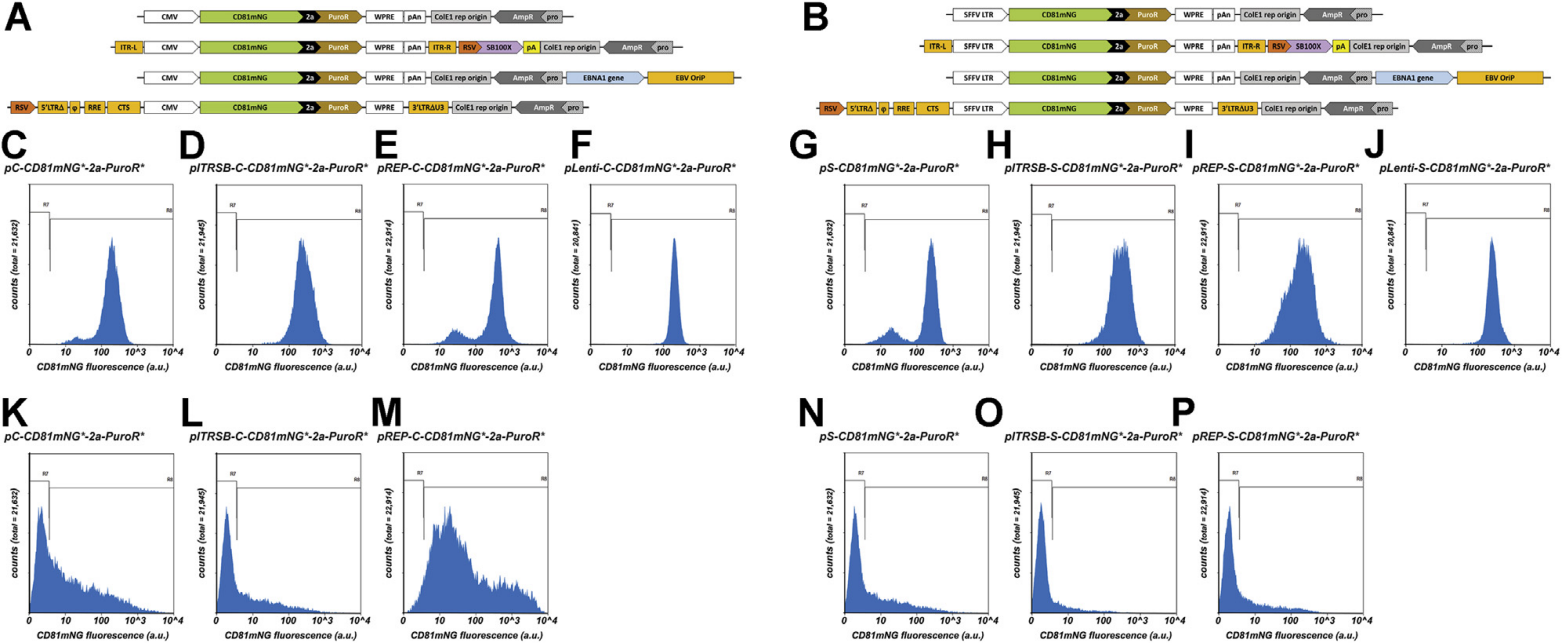
**Effect of transcriptional control elements and mode of transgene delivery on CD81mNG expression.***A* and *B*, line diagrams of plasmid, Sleeping Beauty transposon, EBV-based episome, and lentiviral vectors carrying the (*A*) CMV-CD81mNG-2a-Puro and (*B*) SFFV LTR-CD81mNG-2a-Puro transgenes. *C–J*, HEK293 cells were transfected or transduced with each of these vectors, selected in puromycin, grown in selective media for 4 weeks, and assayed for mNeonGreen fluorescence by flow cytometry. Numbers of cells are shown on the *y*-axis while relative fluorescent brightness (arbitrary units (a.u.)) is shown on the *x*-axis (log scale). *K–P*, HEK293 cells were transfected with the six plasmid vectors shown (*A*, *B*), grown for 2 days in normal media and assayed for mNeonGreen fluorescence by flow cytometry. Numbers of cells are shown on the *y*-axis while relative fluorescent brightness (arbitrary units (a.u.)) is shown on the *x*-axis (log scale). R7 shows the experimentally determined background fluorescence of HEK293 control cells, whereas R8 denotes green fluorescence above background. These experiments were performed twice.

These observations support a working hypothesis in which selectable markers establish a threshold of transgene expression below which no cell can survive. To determine whether this threshold was affected by the mode of transgene delivery, we created a series of eight DNA vectors that express the identical recombinant protein (CD81mNG-2a-PuroR) from two different transcriptional control elements (CMV (9) or SFFV long terminal repeat (LTR) (8)) delivered by four distinct vector systems: nonreplicating plasmids (pC and pS), Sleeping Beauty transposons (pITRSB-C and pITRSB-S) (18, 19), EBV-based episomes (pREP-C or pREP-S) (22), or replication-defective, self-inactivating lentiviruses (Lenti-C or Lenti-S) (Fig. 7, *A* and *B*). HEK293 cells were transfected with the six naked DNA vectors and transduced with the two lentiviral vectors, followed by selection of puromycin-resistant clones to create eight polyclonal cell lines. These were examined by flow cytometry (Fig. 7, *C*–*J*; Table 4), revealing that all eight cell lines displayed roughly similar CD81mNG fluorescence profiles.

**Table 4:** Flow cytometry data for puromycin-resistant HEK293 cells transfected or transduced with different vector systems

Cell line	Average relative brightness; c.v.	% nonexpressing cells
HEK293/pC-CD81mNG^a^-2a-PuroR^a^	202; 55	>0.01%
HEK293/pITRSB-C-CD81mNG^a^-2a-PuroR^a^	294; 60	>0.01%
HEK293/pREP-C-CD81mNG^a^-2a-PuroR^a^	364; 68	0.04%
HEK293/pLenti-C-CD81mNG^a^-2a-PuroR^a^	212; 29	0.01%
HEK293/pS-CD81mNG^a^-2a-PuroR^a^	216; 60	0.40%
HEK293/pITRSB-S-CD81mNG^a^-2a-PuroR^a^	355; 62	0%
HEK293/pREP-S-CD81mNG^a^-2a-PuroR^a^	253; 96	0.03%
HEK293/pLenti-S-CD81mNG^a^-2a-PuroR^a^	273; 41	>0.01%

a Codon optimized.

To test whether similar results would be observed during a short-term, transient expression in the absence of puromycin selection, we transfected HEK293 cells with the same six DNA vectors and measured CD81mNG expression at 2 days posttransfection (Fig. 7, *K*–*P*; Table 5). As expected, these transiently transfected cell populations contained many more nonexpressing cells, a much lower average level of CD81mNG fluorescence, and much higher cell-to-cell variation in CD81mNG expression. Furthermore, these results demonstrated that CMV-based expression was far higher than SFFV LTR-based expression from episomal vectors. These results support our conclusion that the similarity in CD81mNG in expression seen in the eight puromycin-selected cell lines was due to the restrictive nature of puromycin selection. We also collected data on the time course of unselected, CMV-driven, CD81mNG expression, which confirmed that transgene expression declines rapidly in the absence of antibiotic selection from the gene delivery platforms that we used in this study (supporting information).

**Table 5:** Flow cytometry data for transiently transfected HEK293 cell populations

Cell line	Average relative brightness; c.v.	% nonexpressing cells
HEK293/pC-CD81mNG^a^-2a-PuroR^a^	86; 355	37%
HEK293/pITRSB-C-CD81mNG^a^-2a-PuroR^a^	37; 453	59%
HEK293/pREP-C-CD81mNG^a^-2a-PuroR^a^	330; 253	3.8%
HEK293/pS-CD81mNG^a^-2a-PuroR^a^	37; 453	59%
HEK293/pITRSB-S-CD81mNG^a^-2a-PuroR^a^	9.3; 459	81%
HEK293/pREP-S-CD81mNG^a^-2a-PuroR^a^	20; 366	68%

a Codon optimized.

#### Parallel results in monkey COS7 cells

**Figure 8:**
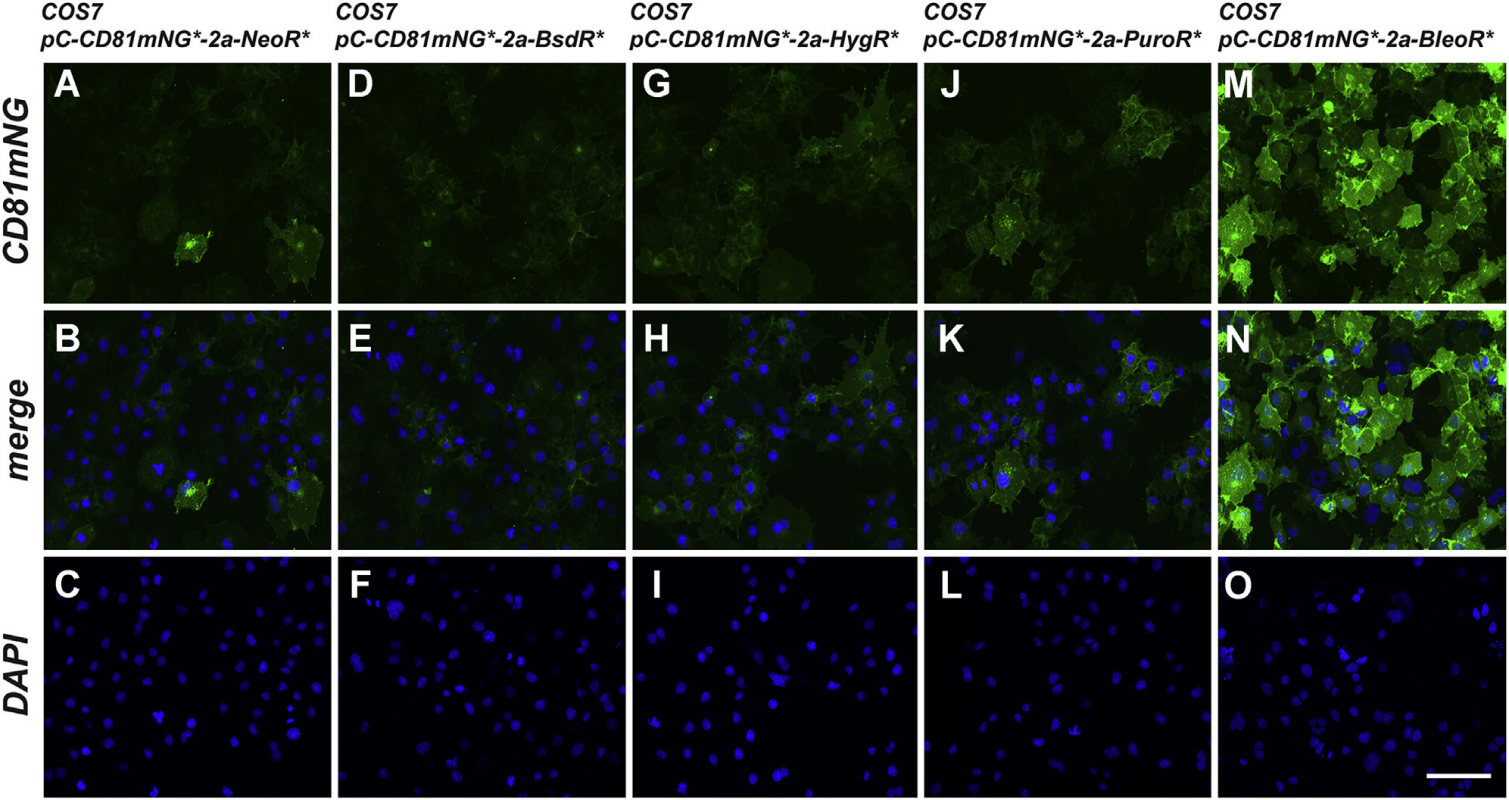
**Fluorescence micrographs of COS7 cell lines carrying CD81mNG-expressing transgenes.** COS7 cells transfected with the five transgenes described in Figure 3*A* were selected for 4 weeks in (*A–C*) G418, (*D–F*) blasticidin, (*G–I*) hygromycin, (*J–L*) puromycin, or (*M–O*) zeocin, respectfully. Each of these five cell lines were then grown overnight on sterile cover glasses, fixed, stained with DAPI. Images show (*A*, *D*, *G*, *J*, *M*) mNeonGreen fluorescence, (*B*, *E*, *H*, *K*, *N*) DAPI fluorescence, and (*C*, *F*, *I*, *L*, *O*) the merge of the two. Bar: 100 μm. These images were selected from three technical replicates of the experiment.

To determine whether choice of selectable marker has a similar effect on transgene expression in other mammalian cell lines, we transfected the simian virus 40-transformed African green monkey kidney cell line COS-7 with the nonreplicating plasmids designed to express CD81mNG from polycistronic ORFs linked by a 2a peptide to the *NeoR* (3) *BsdR* (29), *HygR* (27), *PuroR* (26), and *BleoR* (28) markers (Fig. 2*A*). Polyclonal cell lines were generated from each population of transfected cells by culturing them in their cognate antibiotic for 10–14 days, pooling clones from each transfection, and then expanding them under selection for another 1–2 weeks. These cell lines were then stained and examined by fluorescence microscopy, which revealed the same pattern of linked CD81mNG expression: highest in the cell lines selected in zeocin, lowest in cell lines selected with blasticidin or G418, and intermediate in cell lines selected with hygromycin or puromycin (Fig. 8).

## Discussion

The creation of transgenic mammalian cells is a critical step in many biomedical research projects. However, there is no simple, inexpensive, and rapid method for generating transgenic cell lines that express high and relatively homogeneous levels of linked recombinant proteins. Here we explored the impact that the choice of selectable marker has on the levels of a linked recombinant protein and found that it can have up to a 10-fold effect on expression level. Moreover, the choice of selectable marker also has a pronounced effect on the cell-to-cell variation in transgene expression, with highest variation correlating with the lowest average expression and the lowest cell-to-cell variation observed in the highest-expressing polyclonal cell lines.

The simplest interpretation of these observations is that each selectable marker–antibiotic pair establishes a threshold of transgene expression below which no cell can survive. We anticipate that these thresholds are determined, at least in part, by each marker protein’s mechanism of action, intrinsic activity, and stability within the cell. Given that these variables are likely to be distinct for nearly all proteins, it is not surprising that each marker/antibiotic pair established a distinct threshold of transgene expression. This model posits that highly efficient and long-lived selectable marker proteins inactivate their cognate antibiotic even at very low levels of expression. As a result, use of such markers results in the survival of cells that express almost any level of the linked recombinant protein, which manifests in pooled, polyclonal cell lines that exhibit a low average level of transgene expression and a high degree of cell-to-cell variation in transgene expression levels. These properties correspond relatively well to those we observed for cell lines generated using the *NeoR* or *BsdR* markers, indicating that the *NeoR* and *BsdR* selectable marker enzymes may be highly active, stable, or both. As for the higher levels of transgene expression in hygromycin-resistant or puromycin-resistant cell lines, we posit that the *HygR* and *PuroR* enzymes may be less active, less stable, or both. And finally, the fact that the *BleoR* marker consistently yielded cell lines with the highest and least heterogeneous levels of transgene expression indicates that it has the lowest activity of all selectable marker proteins. This supposition is consistent with its noncatalytic mechanism of zeocin inactivation, as the BleoR protein does not chemically modify zeocin, but rather inactivates it by sequestering it in complexes that have a 1:1 molar ratio of drug to protein (39).

The preceding considerations provide a conceptual framework for understanding how choice of selectable marker is likely to affect transgene expression levels. As for how to make practical use of our findings, our data suggest the following roadmap for rapid creation of polyclonal cell lines that express high levels of a recombinant protein of interest:

(i) Clone the coding region for the protein of interest upstream of, and in frame with, the viral 2a peptide and the *BleoR* marker, with the entire bicistronic ORF inserted downstream of SFFV enhancer/promoter and upstream of the WPRE and pAn sequences of the pITRSB Sleeping Beauty transposon vector.
(ii) Transfect the resulting plasmid into the cell line of interest.
(iii) Two days later, place the cells in zeocin-containing media.
(iv) Change media every 3–4 days for 10–14 days, or however long it takes for all mock-transfected cells to die.
(v) Pool all zeocin-resistant cell clones to create a mixed clone cell line.
(vi) Use this cell line for protein expression, functional analysis, production of purified protein, and/or production of recombinant exosomes.

Additional considerations include the following:

• The vector DNA should be transfected into a large number of actively growing cells (10^7^, or even more) to generate a large number of initial zeocin-resistant SCCs, reducing the amount of time required to obtain a large number of transgenic cells. This requires transfection with a large amount of the vector, 35 μg or more.
• The upstream protein of interest will be expressed with the 17 amino acid-long ‘2a peptide tag’ at its C-terminus, which can be used for protein detection and purification purposes using an anti-2a tag antibody.
• Additional purification and/or detection tags can easily be incorporated by inclusion into the ORF prior to insertion into our expression vectors.
• High efficiency of bicistronic, 2a peptide-based expression systems typically requires an unstructured C-terminus of the upstream protein; otherwise, the proteins may be expressed as a single continuous polypeptide rather than as two separate polypeptides. This can also be added in the vector design, and use of our vectors is designed to add as few as four amino acids (GRSP) or as many as 15 amino acids (GRSPGLNTRLEVGSG) between the upstream protein of interest and the p2a peptide.
• For difficult-to-express proteins, one may not be able to obtain any zeocin-resistant clones, and if this occurs one may need to switch to PuroR- or HygR-based vectors that allow survival at lower levels of transgene expression.
• Although not explored in this study, an inverted position of the selectable marker and protein of interest may also be useful for driving high-level expression of recombinant proteins, especially if the C-terminus of the protein has to be unmodified. However, this arrangement of marker and protein of interest would theoretically allow the survival of some cell clones that may fail to express the entire downstream protein of interest.
• It should be noted that use of the *BleoR* marker and zeocin has the potential to affect cell physiology. Zeocin kills cells by binding DNA and inducing DNA damage (40), and zeocin-induced DNA damage can even occur in *BleoR*-expressing, zeocin-resistant cell lines (41). However, similar concerns also exist for other antibiotics used to select transgenic mammalian cell lines.

**Figure 9:**
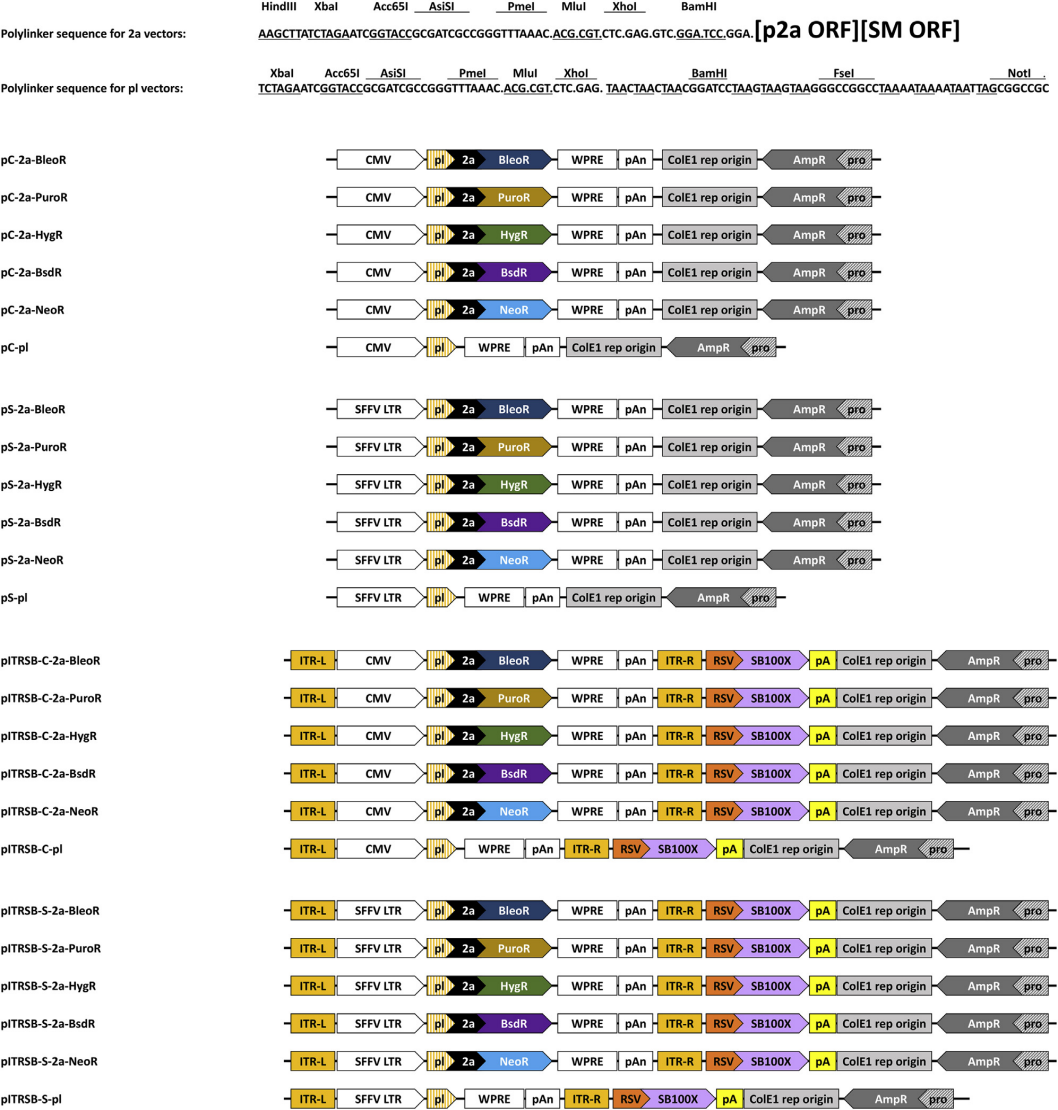
**Line diagrams of nonreplicating and Sleeping Beauty expression vectors.** The top two lines show the DNA sequence of the polylinkers common to all p2a-containing and all pl-designated vectors. The linear plasmid maps depict the relative positions of major design elements of the circular plasmids we created, with the pC plasmids showing nonreplicating vectors with the CMV transcriptional control sequences, the pS plasmids showing the nonreplicating vectors with the SFFV LTR, the pITRSB-C plasmids showing the Sleeping Beauty vectors with the CMV transcriptional control elements, and the pITRSB-S showing the Sleeping Beauty vectors with the SFFV LTR.

Our findings are also likely relevant to the isolation of SCCs that express the very highest levels of linked recombinant proteins. Specifically, use of the BleoR marker is likely to be an effective “pre-screen,” limiting the initial SCCs to those that express the very highest levels of the transgene, thereby reducing the number of SCCs that need to be screened in the search for a cell line that expresses high levels of the protein of interest. As for the other markers, their use is associated with the survival of SCCs that express lower levels of the transgene, requiring the screening of more SCCs. This is particularly true for the *NeoR* marker, which was the first widely used dominant selectable marker gene and is still present in more Addgene plasmids than any other selectable marker. It may be impossible to know how much time, effort, and resources have been wasted in mammalian transgenesis-related projects due to use of this particular selectable marker, but we can hopefully minimize future waste by making vectors available that incorporate the findings of this study. Toward this end, we have assembled a suite of plasmid and Sleeping Beauty transposon vectors that are designed on the principles we have discovered (Fig. 9).

Most of the experiments in this study used CD81 as the test protein of interest, as one of the main goals of this study was to understand the factors that affect exosome engineering. Our experiments revealed that the percentage of exosomes containing a given exosomal cargo is proportional to the levels of cargo protein expression. This observation is consistent with the idea that exosome biogenesis is a stochastic process in which the content of any individual exosome is determined by the levels of exosome cargo molecules in the vicinity of the exosome budding site (30). Moreover, our results demonstrate that the choice of selectable marker is an important consideration in the genetic modification of exosome content, with obvious implications for the production of exosome-based therapies, standards, and controls.

## Experimental procedures

### Plasmids and viruses

Plasmids were maintained in DH10B cells, grown in ampicillin-containing LB media, and purified from bacterial lysates using mini-prep and midi-prep plasmid isolation kits (Promega). DNA sequence data were assembled, maintained, and analyzed using SnapGene software. The 3xNLS-tdTomato, CD81mNG, *BleoR*, *PuroR*, *HygR*, *BsdR*, *NeoR*, and SB100X coding regions were codon-optimized for expression in human cells, synthesized *in vitro*, cloned into mammalian cell expression vectors, and sequence-confirmed prior to use. The ITR-left and ITR-right backbone of the Sleeping Beauty vectors (18, 19) and the *cis*-acting elements of a third-generation, replication-defective and self-inactivating lentiviral backbone (42) were also synthesized *in vitro*, cloned into minimal bacterial plasmids, and sequence-confirmed prior to use. The EBNA1 and OriP sequences (22) were assembled by a combination of *in vitro* gene synthesis and excision from pCEP4 (ThermoFisher), cloned into a minimal plasmid vector, with sequence confirmation of all newly synthesized segments of DNA. Transgenes and polylinkers were inserted into transgene delivery vectors using standard recombinant DNA cloning techniques. Sequences of all vectors used in this study (Table 6) or described in this study (Table 7) are available upon request.

**Table 6:** List of plasmids used in this study

Plasmid name	Plasmid code	Vector type	Enhancer/Promoter
pcDNA3-3xNLS-tdTomato^a^-2a-BsdR^a^	pJM825	Nonreplicating plasmid	CMV
pCJM-3xNLS-tdTomato^a^-2a-NeoR^a^	pJM1074	Nonreplicating plasmid	CMV
pCJM-3xNLS-tdTomato^a^-2a-BsdR^a^	pJM908	Nonreplicating plasmid	CMV
pCJM-3xNLS-tdTomato^a^-2a-HygR^a^	pJM912	Nonreplicating plasmid	CMV
pCJM-3xNLS-tdTomato^a^-2a-PuroR^a^	pJM916	Nonreplicating plasmid	CMV
pCJM-3xNLS-tdTomato^a^-2a-bleoR^a^	pJM904	Nonreplicating plasmid	CMV
pC-CD81mNG^a^-2a-NeoR^a^	pCG18	Nonreplicating plasmid	CMV
pC-CD81mNG^a^-2a-BsdR^a^	pCG14	Nonreplicating plasmid	CMV
pC-CD81mNG^a^-2a-HygR^a^	pCG16	Nonreplicating plasmid	CMV
pC-CD81mNG^a^-2a-PuroR^a^	pCG10	Nonreplicating plasmid	CMV
pC-CD81mNG^a^-2a-BleoR^a^	pCG12	Nonreplicating plasmid	CMV
pS-CD81mNG^a^-2a-PuroR^a^	pJM1277	Nonreplicating plasmid	CMV
pITRSB-C-CD81mNG^a^-2a-PuroR^a^	pJM1358	Sleeping Beauty transposon	CMV
pITRSB-S-CD81mNG^a^-2a-PuroR^a^	pJM1366	Sleeping Beauty transposon	CMV
pREP-C-CD81mNG^a^-2a-PuroR^a^	pCG43	Replicating episome	CMV
pREP-S-CD81mNG^a^-2a-PuroR^a^	pJM1367	Replicating episome	CMV
pLenti-C-CD81mNG^a^-2a-PuroR^a^	pJM1291	Lentiviral provirus	CMV
pLenti-S-CD81mNG^a^-2a-PuroR^a^	pJM1293	Lentiviral provirus	CMV

a Codon optimized.

**Table 7:** Vector series for transgene expression

Plasmid name	Plasmid code	Vector type	Enhancer/Promoter
pC-2a-BleoR^a^	pJM1245	Nonreplicating plasmid	CMV
pC-2a-PuroR^a^	pJM1242	Nonreplicating plasmid	CMV
pC-2a-HygR^a^	pJM1247	Nonreplicating plasmid	CMV
pC-2a-BsdR^a^	pJM1246	Nonreplicating plasmid	CMV
pC-2a-NeoR^a^	pJM1248	Nonreplicating plasmid	CMV
pC-pl	pJM1329	Nonreplicating plasmid	CMV
pS-2a-BleoR^a^	pJM1345	Nonreplicating plasmid	SFFV LTR
pS-2a-PuroR^a^	pJM1344	Nonreplicating plasmid	SFFV LTR
pS-2a-HygR^a^	pJM1346	Nonreplicating plasmid	SFFV LTR
pS-2a-BsdR^a^	pJM1400	Nonreplicating plasmid	SFFV LTR
pS-2a-NeoR^a^	pJM1347	Nonreplicating plasmid	SFFV LTR
pS-pl	pJM1330	Nonreplicating plasmid	SFFV LTR
pITRSB-C-2a-BleoR^a^	pJM1384	Sleeping Beauty transposon	CMV
pITRSB-C-2a-PuroR^a^	pJM1355	Sleeping Beauty transposon	CMV
pITRSB-C 2a-HygR^a^	pJM1385	Sleeping Beauty transposon	CMV
pITRSB-C-2a-BsdR^a^	pJM1401	Sleeping Beauty transposon	CMV
pITRSB-C-2a-NeoR^a^	pJM1386	Sleeping Beauty transposon	CMV
pITRSB-C-pl	pJM1356	Sleeping Beauty transposon	CMV
pITRSB-S-2a-BleoR^a^	pJM1389	Sleeping Beauty transposon	SFFV LTR
pITRSB-S-2a-PuroR^a^	pJM1388	Sleeping Beauty transposon	SFFV LTR
pITRSB-S-2a-HygR^a^	pJM1390	Sleeping Beauty transposon	SFFV LTR
pITRSB-S-2a-BsdR^a^	pJM1402	Sleeping Beauty transposon	SFFV LTR
pITRSB-S-2a-NeoR^a^	pJM1391	Sleeping Beauty transposon	SFFV LTR
pITRSB-S-pl	pJM1393	Sleeping Beauty transposon	SFFV LTR

a Codon optimized.

To make replication-defective, self-inactivating lentiviruses, 293T cells were transfected with a mixture of four plasmids: the lentiviral vector, a Gag-Pol expression vector, a Rev expression vector, and a VSV-G expression vector (42). The transfected cells were incubated for 3 days. The tissue culture supernatant was collected, spun at 5000*g* for 15 min to remove cells and cell debris, and the resulting supernatant was passed through a 0.45 μm filter to generate an unconcentrated virus stock.

### Cell culture, transfection, transduction, and antibiotic selection

All cell lines were grown in a tissue culture incubator maintained at 5% CO_2_, 90–99% humidity, and 37 °C. HEK293 and 293T cells were obtained from the American Type Culture Collection (ATCC) and grown in DMEM (high glucose; Gibco/BRL) supplemented with 10% fetal calf serum (FCS; ThermoFisher). COS7 was a generous gift from Dr Natasha Zachara and was also grown in DMEM (high glucose; Gibco/BRL) supplemented with 10% fetal calf serum (FCS; ThermoFisher). 293F cells (ThermoFisher) were grown either as suspension cells in chemically defined Freestyle media (ThermoFisher) in uncoated tissue culture shaker flasks on a shaking platform at 110 rpm or in DMEM supplemented with 10% FCS in standard, coated tissue culture flasks or dishes. Cells were transfected using Lipofectamine 2000 reagent (ThermoFisher). In brief, 5 μgs plasmid DNA was diluted into 0.3 ml of Opti-Mem medium (Gibco/BRL), while 15 μl of Lipofectamine 2000 was diluted into a separate 0.3 ml of Opti-Mem medium, and both mixtures were incubated separately for 5 min. The two mixtures were mixed together and incubated for a further 15 min. Next, growth medium was removed from a T-25-coated tissue culture flask containing HEK293 cells at ~70–90% confluency. The cells were then washed with 5 ml of Opti-Mem medium (at 37 °C), all liquid was removed, and the 0.6 ml mixture of DNA, Lipofectamine 2000, and Opti-Mem was added to the flask. After gentle rocking to distribute the mixture across the entire flask surface, the flask was incubated for 15–20 min in a tissue culture incubator. The DNA/Lipofectamine 2000/Opti-MEM solution was then removed, 5 ml DMEM +10% FCS was added to each flask, and the cells were then returned to the incubator for between 1 and 2 days, depending on the experiment. For lentiviral transductions, 1 ml of unconcentrated virus stock was added to 10 ml of culture media in the presence of 6 μg/ml polybrene and ~3 × 10^6^ cells in a 10 cm tissue culture dish, incubated for 2 days, and then washed.

Antibiotic-resistant cell lines were generated from transfected or transduced cell HEK293 cells by splitting cells onto 150 mm dishes containing DMEM, 10% FCS, and the appropriate antibiotic. Antibiotics were used at the following concentrations: 400 μg/ml G418, 20 μg/ml blasticidin, 400 μg/ml hygromycin B, 3 μg/ml puromycin, and 200 μg/ml zeocin, concentrations that are within the range suggested by the manufacturer for this cell line (Invivogen, Inc.). Transfected cell populations were refed every 3–4 days until distinct, drug-resistant clones were large enough to be seen by the eye, and all antibiotic-sensitive cells between the drug-resistant colonies had died off, typically 10–14 days. The drug-resistant cells from each transfected population were then pooled to create polyclonal cell lines and expanded until they had grown for 4 weeks from the date of transfection in selective media. Each cell line was then processed for flow cytometry, fluorescence microscopy, and/or immunoblot.

### Flow cytometry

Cells were suspended by trypsinization, washed in Hank’s buffered saline solution (HBSS), and resuspended at a concentration of 1 × 10^7^ cells per ml in cold (4 °C) HBSS containing 0.1% FBS. Cell suspensions were maintained on ice, diluted to a concentration of 1 × 10^6^ cells per ml, and examined for tdTomato or mNeonGreen fluorescence by flow cytometry on a Beckman MoFlo Cell Sorter equipped with 355 nm, 488 nm, and 633 nm lasers set to the appropriate detection wavelength. The relative brightness was determined for thousands of individual cells in each cell line using Beckman MoFlo software and reported as scatter plots, average relative brightness, and coefficient of variation,

### Immunofluorescence

Cells were seeded onto sterile (autoclaved) borosilicate cover glasses in tissue culture dishes and grown overnight in normal media. The cover glasses were removed from the tissue culture dishes, washed, and fixed in 3.7% formaldehyde in Dulbecco’s modified phosphate-buffered saline (DPBS), pH 7.4, for 15 min. The cover glasses were then washed in DPBS, incubated in DPBS containing DAPI (5 μg/ml) for 15 min, washed five times in DPBS, and mounted on a glass slide containing ~8 μl of mounting solution (90% glycerol, 100 mM Tris pH8.5, 0.01% *para*-phenylenediamine). After removal of excess mounting solution, the cells were examined using a Nikon Eclipse TE200 microscope equipped with Nikon S Fluor 20×, 0.75 aperture objective and an Andor Neo sCMOS DC-152Q-C00 F digital camera. Images were processed using Photoshop and assembled in illustrator (Adobe).

### Immunoblot

Equal numbers of each HEK293-derived cell line were lysed in SDS-PAGE sample buffer (20% glycerol, 4% SDS, 120 mM Tris-HCl (pH 6.5), 0.02% bromophenol blue) at room temperature, then frozen, and thawed once. The thawed samples were then boiled for 10 min, spun at 10,000*g* for 2 min to pellet insoluble materials, loaded onto 4–15% polyacrylamide gradient gels (Bio-Rad), and electrophoresed according to the manufacturer’s suggestions. Proteins were then transferred to immobilon-P membranes (Amersham), incubated in blocking solution (5% nonfat dry milk in TBST (138 mM NaCl, 2.7 mM KCl, 50 mM Tris, pH 8.0, 0.05% Tween-20) for 2 h, and then in blocking solution containing primary antibodies overnight at 4 °C (rabbit polyclonal anti-p2a antibody was used at a dilution of 1:1000 and anti-actin antibodies were used at a dilution of 1:1000). The membranes were washed five times in TBST and then incubated with blocking solution containing secondary antibodies conjugated with horseradish peroxidase (HRP) at a dilution of 1:5000 for 1 h. The membranes were washed five times with TBST, incubated in HRP-activated chemiluminescence detection solution (Amersham ECL Western Blotting Detection Reagents; cat# RPN2106), and imaged using a GE Amersham Imager 600. Images were exported as JPEG files, analyzed using ImageJ software, and processed using Photoshop software (Adobe).

### Exosome analysis

293F-derived cells were grown in sterile shaker flasks containing 80 ml of Freestyle media for 5 days at a starting concentration of 5 × 10^5^ cells/ml. We then generated clarified tissue culture supernatants (CTCSs) of each culture by centrifuging the cultures at 5000*g* for 15 min to remove all cells and passing the resulting supernatant through a 0.22 μm filter to remove large cell debris. The ~80 mls of CTCS was then concentrated to ~0.5 ml using a 100 kDa molecular weight cutoff angular filtration unit (Centricon-70) according to the manufacturer’s suggestions. The resulting samples were then passed over an Izon qEV 35 nm size-exclusion chromatography column using PBS, pH 7.4 as column buffer, and collecting 0.5 mls fractions. Fractions 4, 5, and 6 contain exosomes (data not shown) and were pooled to generate each exosome preparation. These were examined by nanoparticle tracking analysis using a Particle Matrix ZetaView Twin 488 & 640 (PMX-220-12C-R4) according to the manufacturer’s suggestions.

## Data availability

All data are contained within this article.

## Ethics Statement

All work was performed according to Johns Hopkins University regulations regarding use of recombinant DNA and human cell lines.

## Supporting information

This article contains supporting information.

## Conflict of interest

F. K. F., C. G., S. J. T, and S. J. G. are coinventors of materials described in this report that are owned by Johns Hopkins University, and if licensed for commercial uses will return royalties to each. S. J. G. currently holds equity in companies that may potentially benefit from the information described in this report, either indirectly or directly.
