# Supplementary material for: Choice of selectable marker affects recombinant protein expression in cells and exosomes
Source: J Biol Chem. 2021 May 27;297(1):100838. doi: 10.1016/j.jbc.2021.100838 (PMC8258971; doi:10.1016/j.jbc.2021.100838)
Supplement: Supporting information [file mmc1.pdf]

Supporting Information

I.

Table S1. Quantitative analysis of immunoblot band intensity from Figure 5. Band intensities were quantified from immunoblot images using Image J followed by statistical analysis of band intensity using Prism.

**Absolute expression (a.u.) CD81**					
**biological rep**	**NeoR**	**BsdR**	**HygR**	**PuroR**	**BleoR**
**#1**	107741	88003	278561	487901	691469
**#2**	64297	50364	199763	419885	705205
**#3**	94008	66007	226956	492653	728867
**average**	88682	68125	235093	466813	708514
**stdev**	22206	18909	40024	40710	18917
**t-test**		0.2908	0.0104	0.0007	< 0.0001
**significance**		ns	*	***	****

**Relative expression CD81/actin**					
**biological rep**	**NeoR**	**BsdR**	**HygR**	**PuroR**	**BleoR**
**#1**	0.27	0.22	0.88	1.93	3.73
**#2**	0.19	0.14	0.53	1.30	2.31
**#3**	0.23	0.16	0.54	1.47	1.99
**average**	0.23	0.17	0.65	1.57	2.67
**stdev**	0.04	0.04	0.20	0.33	0.93
**t-test**		0.153	0.0618	0.0186	0.0444
**significance**		ns	ns	*	*

II.

Time course of decline in unselected expression, by day.

To better understand the effect of antibiotic selection on transgene expression, we followed the expression of CD81mNG in HEK293 cells following transfection with the plasmids pREP-C-CD81mNG-2a-PuroR, pC-CD81mNG-2a-PuroR, and pITRSB-C-CD81mNG-2a-PuroR, but without adding puromycin to the culture media. These three cell populations were assayed by fluorescence microscopy on days 2, 3, 4, 7, 10, and 14 (***Figure S1***) and by flow cytometry on days 2, 3, 4, 5, and 6 after transfection by flow cytometry (***Figure S2***). The results of these experiments confirm the previously-established fact that transgene expression at two days after transfection is high and falls on each passing day. Expression from the replicating (pREP) vector was highest but unstable, expression from the non-replicating (pC) vector was lowest, and expression from the

Sleeping Beauty transgene was initially lowest but rose above the pC-generated population over time.

Figure S1. Fluorescence microscopy confirms that transgene expression declines rapidly over time in the absence of antibiotic selection. Fluorescence micrographs showing CD81mNG expression in HEK293 cells on days 2, 3, 4, 7, 10, and 14 after transfection with a replicating plasmid (pREP-C-CD81mNG-2a-PuroR), a non-replicating plasmid (pC-CD81mNG-2a-PuroR), and a Sleeping Beauty vector (pITRSB-C-CD81mNG-2a-PuroR). Bar, 100 µm. These images were selected from three technical replicates of the experiment.

Figure S2. Flow cytometry confirms that transgene expression declines rapidly over time in the absence of antibiotic selection. Flow cytometry measurements of CD81mNG fluorescence in HEK293 cells on days 2, 3, 4, 5, and 6 after transfection with a replicating plasmid (pREP-C-CD81mNG-2a-PuroR), a non-replicating plasmid (pC-CD81mNG-2a-PuroR), and a Sleeping Beauty vector (pITRSB-C-CD81mNG-2a-PuroR). These results are from three technical replicates.

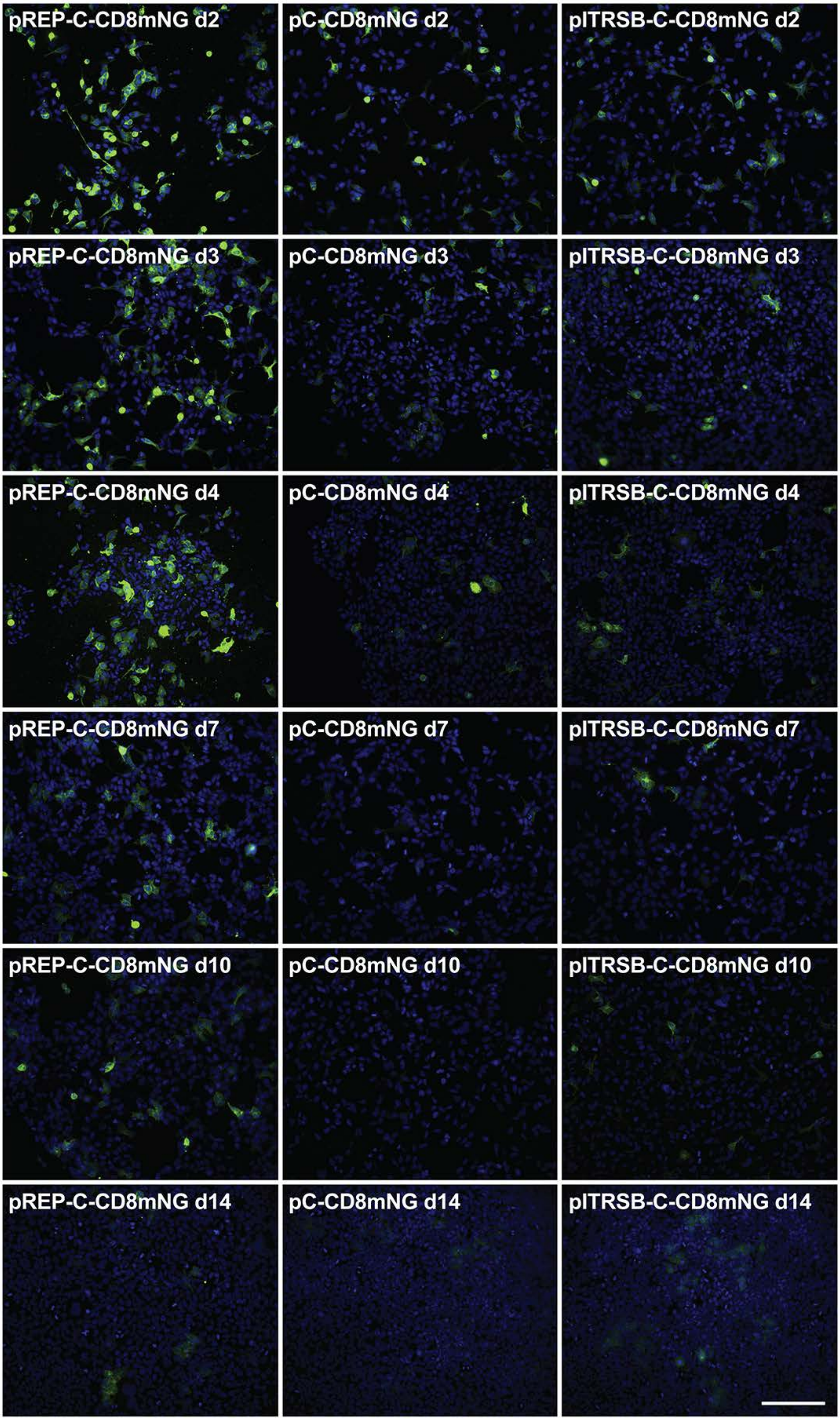
pREP-C-CD8mNG d2
pC-CD8mNG d2
pITRSB-C-CD8mNG d2
pREP-C-CD8mNG d3
pC-CD8mNG d3
pITRSB-C-CD8mNG d3
pREP-C-CD8mNG d4
pC-CD8mNG d4
pITRSB-C-CD8mNG d4
pREP-C-CD8mNG d7
pC-CD8mNG d7
pITRSB-C-CD8mNG d7
pREP-C-CD8mNG d10
pC-CD8mNG d10
pITRSB-C-CD8mNG d10
pREP-C-CD8mNG d14
pC-CD8mNG d14
pITRSB-C-CD8mNG d14

Figure S1

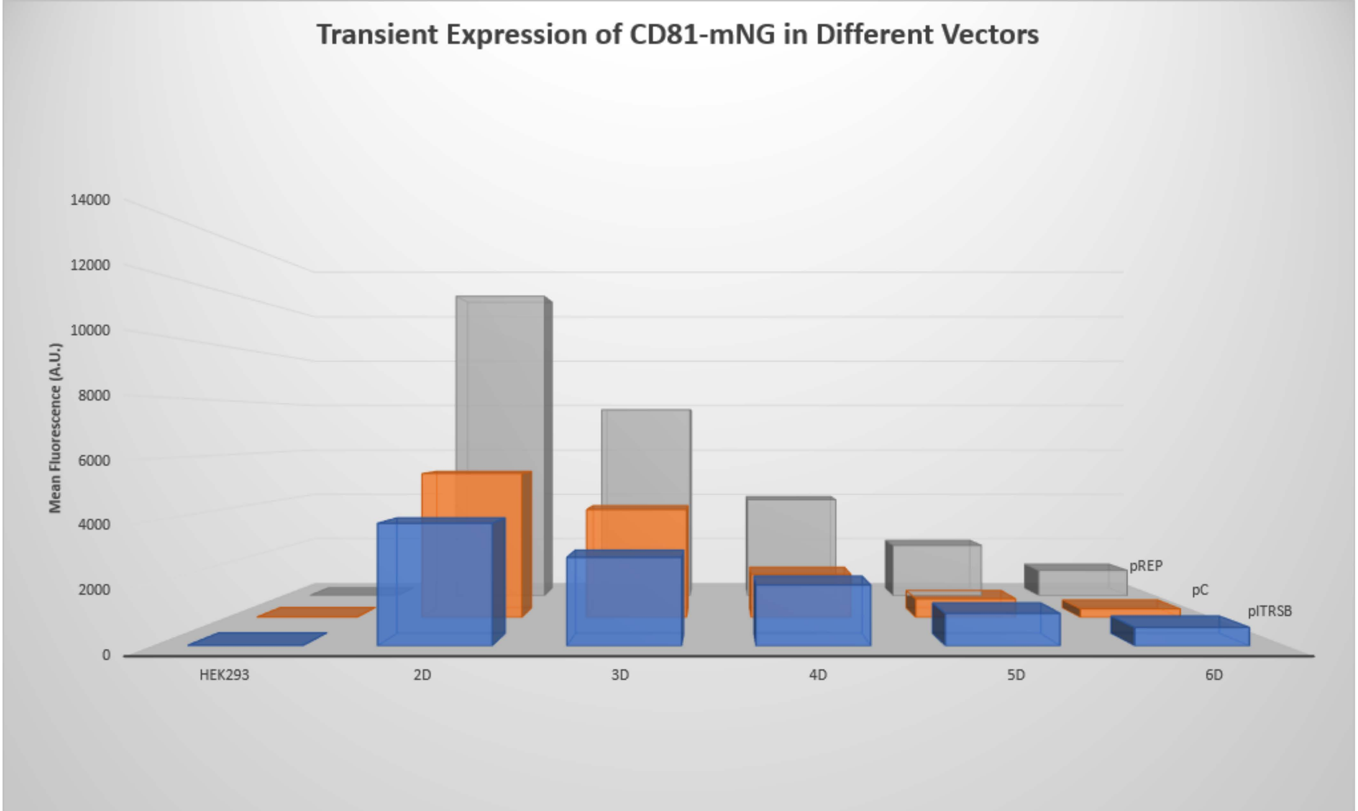

	HEK293	2d	3d	4d	5d	6d
pITRSB	24	3934	2840	1953	1042	595
pC	24	5309	3978	1587	686	316
pREP	24	12480	7753	3998	2093	1051

pITRSB pC pREP

Figure S2 Guo et al.
